# Supplementary material for: PET-measured human dopamine synthesis capacity and receptor availability predict trading rewards and time-costs during foraging
Source: Nat Commun. 2023 Sep 30;14:6122. doi: 10.1038/s41467-023-41897-0 (PMC10542376; doi:10.1038/s41467-023-41897-0)
Supplement: Supplementary file 1 — Supplementary Information [file 41467_2023_41897_MOESM1_ESM.pdf]

PET-Measured Human Dopamine Synthesis Capacity and Receptor Availability Predict Trading Rewards and Time-Costs during Foraging

Supplementary Text

Additional Behavioral Results
- Optimal Leaving Thresholds
- Dynamics of Average Leaving Threshold within Reward Environment
- Behavioral Sensitivity to Travel Time and Decay Rate

Additional PET Data Correlations with Foraging Behavior
- PCA Component Correlations with Mean Patch-Leaving Thresholds
- Individual ROI Correlations with Mean Patch-Leaving Threshold
- Individual ROI Correlations with Total Change in Patch-Leaving Threshold
- Individual ROI and PCA Component Correlations with Total Change in Reaction Time
- Individual ROI Correlations with Change in Leaving Threshold due to Travel Time and Decay Rate
- PCA Component Correlations with Leaving Threshold Dynamics
- Correlations with MVT-Predicted Optimal Exit Threshold

Body Mass Index (BMI) Association with Foraging Behavior and PET Measures

Temporal Stability of Results
- Test-Retest Reliability of Foraging Behavioral Measures
- ROI Results Controlling for Time Between Behavioral Task and PET Scan

Reliability of PET PCA Results

Supplementary Figures

Supplementary Figure 1: Moving average reward patch exit threshold over exit decisions, grouped by current reward environment.

Supplementary Figure 2: Moving average reward patch exit threshold over exit decisions, grouped by previous reward environment.

Supplementary Figure 3: Moving average reward patch exit threshold over exit decisions, grouped by current reward environment and colored by prior block that the participant encountered.

Supplementary Figure 4: Difference between current and asymptotic (average of last three exit decisions) exit threshold within each reward patch, grouped by current reward environment.

Supplementary Figure 5: Group mean and standard error of the mean (SEM) of the difference between current and asymptotic (average of last three exit decisions) exit threshold for each exit decision, grouped by current reward environment (block).

Supplementary Figure 6: Difference between current and asymptotic (average of last three exit decisions) exit threshold for each exit decision, group by prior reward environment that the participant experienced.

Supplementary Figure 7. Group mean and standard error of the mean (SEM) of the difference between current and asymptotic (average of last three exit decisions) exit threshold, grouped by prior reward environment that the individual encountered.

Supplementary Figure 8: Stability of change in exit threshold and change in reaction time across two testing dates.

Supplementary Text

Additional Behavioral Results

Optimal Leaving Thresholds

According to the marginal value theorem (MVT), the optimal exit thresholds for leaving a reward patch with the specific environment parameters used in this task were as follows: 5.88 for the long travel time and steep decay rate environment (long steep), 6.56 for the long travel time and shallow depletion rate environment (long shallow), 7.74 for the short travel time and steep depletion rate environment (short steep), and 8.04 for the short travel time and shallow depletion rate environment (short shallow). Since the task parameters were drawn from a beta distribution, the optimal values were calculated as the average result from 100,000 simulations of the task using the criteria defined by the MVT (i.e. that it is optimal to leave the current patch when the predicted reward from the next harvest falls below the average reward rate for the environment). MVT simulations were run in MATLAB.

For the participants who completed this study, the average thresholds for leaving a reward patch generally followed the same pattern as the optimal thresholds (Figure 2: long steep = 4.17, long shallow = 4.77, short steep = 5.30, short shallow = 5.58), however, the averages were consistently lower than the optimal thresholds. Our measure of behavioral sensitivity to the reward environment (i.e. the total change in patch-leaving threshold between the short shallow environment with the highest average reward rate and the long steep environment with the lowest average reward rate) ranged from -2.16 to 5.22, with a mean of 1.41 and standard deviation of 1.83.

Dynamics of Average Leaving Threshold within Reward Environment

Although experimental variables depletion rate and travel time remaining fixed throughout each reward environment, participants were still required to learn about these parameters through sampling when they first entered a new environment. We plotted the running average of the patch leaving threshold over exit decisions within each block and observed that participants did tend to adjust their patch-leaving threshold after the first few decisions before generally settling on a stable threshold for leaving (see Supplementary Figure 1). However, we also noted individual differences such that some participants and reward environments appeared to have greater learning effects than others, which are also modulated by the previous reward environment that the participant encountered (see Supplementary Figures 2 and 3).

To investigate stability of exit thresholds within participants, we ran correlations between the first and final average exit threshold for each subject within each reward environment and found that these were highly correlated, suggesting intra-subject stability in patch-leaving threshold (short-shallow: r=0.663, p=2.59e-9; short-steep: r=0.760, p=1.15e-11; long-shallow: r=0.599, p=1.10e-6; long-steep: r=0.312, p=1.91e-2). Additionally, we calculated the average exit threshold for each subject excluding their first three exit decisions within each block. The overall average leaving threshold with and without these first three exit decisions were also highly correlated (short-shallow environment r=0.970, p=6.78e-35; short-steep environment r=0.977, p=7.17e-38; long-shallow environment r=0.940, p=2.19e-26; long-steep environment r=0.969, p=1.49e-24).

To formally test for learning effects, we used two approaches. First, we assessed the slope of the moving average patch leaving threshold within each reward environment. We used the lm function in R to run a linear regression of average patch leaving threshold over exit decisions within each individual and each reward environment and extracted the coefficient representing the slope of this association. We then ran a t-test across all participants to assess whether the slope was significantly different from zero for each reward environment. We found significant effects of slope in the short-shallow (t=3.688, p=5.178e-4, df=55), long-shallow (t=-2.7129, p=8.886e-3), and long-steep (t=-2.6875, p=9.506e-3) reward environments. The slope in the short-steep reward environment was not significantly different from zero (t=-0.80855, p=0.4223). Assessing longer term learning effects by recalculating the slope of change in average leaving threshold over exit decisions after excluding the first three exit decisions, only the short-shallow reward environment slope remained significantly different from zero (t=2.361, p=2.193e-2) and the remaining reward environments dropped to trends or non-significant effects (short-steep t=0.15199, p=0.8798; long-shallow t=-1.8605, p=0.06859; long-steep t=-0.66973, p=0.5058). These results suggest that participants tended to learn early in the block to appropriately adjust their leaving-threshold towards a higher value in the short-shallow reward environment and towards a lower value in the environments with the long travel time. However, the learning effects in the richest reward environment (short-shallow) tended to persist beyond the initial three exit decisions.

The second approach we used to assess learning effects was to calculate the difference between each individual patch-leaving decision threshold and the asymptotic patch leaving threshold (average of the patch-leaving threshold in the last three decisions) in each reward environment. We plotted these data for individual participants as well as the mean and standard error across the group based on the current reward environment (see Supplementary Figures 4 and 5) and previous reward environment (see Supplementary Figures 6 and 7). Participants tend to adjust their exit threshold towards the asymptotic threshold after the first exit decision, appropriately increasing their threshold in the short-shallow reward environment and decreasing their threshold in the environments with the long travel time (see Supplementary Figure 5). The learning effects of prior block appear to last a few more trials in certain cases, specifically in the first block of the experiment and after the long-steep reward environment (see Supplementary Figure 7). Furthermore, the largest initial adjustment in patch leaving threshold appears to occur in the first reward environment of the experiment and following the short-shallow reward environment (see Supplementary Figure 7).

Behavioral Sensitivity to Travel Time and Decay Rate

Behavioral sensitivity to the travel time and decay rate parameters were quantified as the amount of change in the average patch-leaving thresholds when these parameters were modified. Specifically, the change in threshold due to travel time differences (i.e. average threshold from environments with short travel time minus the average threshold from environments with long travel time) ranged from -2.33 to 4.44,

with a mean of 0.97 and standard deviation of 1.30. The change in threshold due to decay rate differences (i.e. the average threshold from the environments with the shallow depletion rate minus the average threshold from the environments with the steep depletion rate) ranged from -1.92 to 2.83, with a mean of 0.44 and standard deviation of 1.12.

Additional PET Data Correlations with Foraging Behavior

PCA Component Correlations with Mean Patch-Leaving Thresholds

To aid in interpretation of the threshold change results, we also ran linear regressions with mean patch-leaving threshold (across all four reward environments as well as each individually) as the dependent variable and the four dopamine PET PCA component scores as the independent variables. The mean patch-leaving threshold across all reward environments was not related to the dopamine PCA component scores (complete model $p=0.719$, individual component score p-values>0.3). When looking at the mean leaving thresholds for the individual reward environments, we found that the mean threshold for the reward environment with the highest average reward rate (short travel time and shallow decay rate) was positively correlated with component 1 score (t-stat=2.136, $p=0.0404$) although the complete regression model including all PCA components was not significant ($p=0.141$). There were no significant correlations with the PCA component scores and the mean leaving thresholds for any of the other reward environments (all p-values>0.1).

Individual ROI Correlations with Mean Patch-Leaving Threshold

We ran linear partial correlations between each PET ROI value and our behavioral measures of interest, controlling for age and gender. There were no significant correlations between mean patch-leaving threshold across all environments and the individual PET ROI values (all p-values>0.1). When looking at the individual reward environment patch-leaving thresholds, there were significant positive correlations between the leaving-threshold for the short-shallow reward environment and $D_{2/3}$ receptor binding potential in the caudate nucleus (r=0.3141, p=0.0455), ventral striatum (r=0.3216, p=0.0403), and a trend in the putamen (r=0.2958, p=0.0604). There was also a trend towards a positive correlation with D_1 receptor binding potential in the ventral striatum (r=0.2743, p=0.0751). There were no significant correlations between dopamine presynaptic synthesis capacity and any of the individual environment leaving thresholds or between D_1 or $D_{2/3}$ receptor binding potential and the leaving thresholds in any of the other reward environments (short-steep, long-shallow, and long-steep; all p-values>0.1).

Individual ROI Correlations with Total Change in Patch-Leaving Threshold

For the total change in patch-leaving threshold between the most and least rewarding environments, we found a positive correlation with D_1 receptor availability in the ventral striatum (r=0.378, p=0.0123), and trends in the ACC (r=0.286, p=0.0626) and putamen (r=0.274, p=0.0752). In addition, there were positive trends between total change in patch-leaving threshold and $D_{2/3}$ receptor availability in the putamen (r=0.279, p=0.0896), caudate nucleus (r=0.294, p=0.0732), and ventral striatum (r=0.305,

p=0.0623). Lastly, there was a positive trend between total change in patch-leaving threshold and dopamine presynaptic synthesis capacity in the ACC (r=0.251, p=0.0857). No regions were significant after correcting for multiple comparisons across all 14 ROIs tested.

Individual ROI and PCA Component Correlations with Total Change in Reaction Time

With regards to the total change in reaction time between the most and least rewarding environments, we found a positive correlation with D_1 receptor availability in the ventral striatum (r=0.378, p=0.0123) and trends in the ACC (r=0.286, p=0.0626) and putamen (r=0.274, p=0.0752). In addition, we found a positive trend with $D_{2/3}$ receptor availability in the ventral striatum (r=0.300, p=0.0676). Lastly, we found a positive correlation between change in reaction time and presynaptic dopamine synthesis capacity in the midbrain (r=0.409, p=0.0039) with trends in the ACC (r=0.267, p=0.0663) and ventral striatum (r=0.261, p=0.0732). Again, no regions were significant after correcting for multiple comparisons. Given that dopamine may have different effects on reaction time and exit threshold, we ran additional linear correlations between PCA components 2 and 3 and the total change in reaction time. However, there were no significant correlations or trends (minimum p-value=0.246).

Individual ROI Correlations with Change in Leaving Threshold due to Travel Time and Decay Rate

Decomposing the change in leaving threshold down into the effects of travel time and decay rate, we found that the change in threshold due to travel time was positively correlated with D_1 receptor availability in the ACC (r=0.306, p=0.0458) and ventral striatum (r=0.383, p=0.0113) with a trend in the putamen (r=0.285, p=0.0641). The

change in leaving threshold due to travel time was also positively correlated with $D_{2/3}$ receptor availability in the caudate nucleus (r=0.323, p=0.0482) with trends in the putamen (r=0.293, p=0.0745) and ventral striatum (r=0.277, p=0.0920). Lastly, there was a positive trend between the change in leaving threshold due to travel time and presynaptic dopamine synthesis capacity in the ACC (r=0.242, p=0.0972) and midbrain (r=0.245, p=0.0931). None of these correlations were significant after multiple comparison correction. There were no significant correlations or trends between change in leaving threshold due to decay rate and any of the PET ROI values (all p-values>0.1).

PCA Component Correlations with Leaving Threshold Dynamics

To assess whether PET PCA component associations with change in leaving threshold reflects choice policy rather than initial learning dynamics, we recalculated the total change in leaving threshold after excluding the first three leave decisions. We found that the correlations with change in leaving threshold and PCA components 1 and 4 remained significant using the filtered threshold values (component 1 t-stat=2.72, p=1.04e-2; component 4 t-stat = 2.92, p=6.44e-3, degrees of freedom=32). In addition, we ran partial correlations controlling for within block learning dynamics to assess whether the associations between PET PCA components 1 and 4 and the total change in patch leaving threshold between the rich and poor environment were independent from within-block learning effects.

First, we used the slope of the average leaving threshold throughout the entire reward environment block as a control variable. We ran separate partial correlations controlling for the slope in each of the reward environments. We found that the

correlation between PCA component 1 and the total change in patch leaving threshold remained significant even after controlling for the slopes in each of the reward environments (short-shallow: r=0.4114, p=0.0127; short-steep: r=0.4811, p=0.0030; long-shallow: r=0.3954, p=0.0170; long-steep: r=0.3659, p=0.0282). The correlation between PCA component 4 and the total change in patch leaving threshold remained significant when controlling for the slope in the short-shallow (r=0.3568, p=0.0327) and long-shallow (r=0.3969, p=0.0165) reward environments, but dropped to a trend when controlling for the slope in the long-steep reward environment (r=0.2898, p=0.0864) and was no longer significant after controlling for the slope in the short-steep reward environment (r=0.1951, p=0.2542).

Second, we took a complimentary approach to control for within-block learning dynamics by calculating the difference between the first exit threshold and the asymptotic average exit threshold (average of last three exit thresholds) in each block. We then included this signed threshold difference as a control parameter in partial regression analyses measuring the correlation between PET PCA components 1 and 4 and the total change in patch leaving threshold between the rich and poor reward environments. Consistent with the slope approach reported above, we found that the correlation between PCA component 1 and the total change in patch leaving threshold between the rich and poor environments remained significant after controlling for the within-block change in threshold in the short-shallow (r=0.4496, p=0.0059), short-steep (r=0.4277, p=0.0093), and long-shallow (r=0.4007, p=0.0154), and long-steep reward environments (r=0.3453, p=0.0392). For PCA component 4, the total change in patch

leaving threshold remained significant after controlling for the within-block change in threshold in the short-shallow (r=0.3710, p=0.0259) and long-shallow (r=0.3738, p=0.0247) reward environments, but dropped to a trend when controlling for within-block threshold change in the long-steep reward environment (r=0.3193, p=0.0577) and was no longer significant when controlling for within-block change in threshold in the short-steep reward environment (r=0.2231, p=0.1910). Overall, these results suggest that the dopamine PET principal components primarily explain between-block changes in leaving threshold. However, the pattern of dopamine variation in component 4 may also be important for within-block learning, specifically in the reward environments with steep depletion rates.

Correlations with MVT-Predicted Optimal Exit Threshold

For each subject, we calculated the absolute value of the difference between their average exit threshold for each patch and the optimal leaving threshold according to the MVT. We then ran linear correlations with the PET PCA and individual ROI values. There were no significant correlations or trends with the PCA scores (all p-values>0.1). There was a negative trend between the deviation from optimal leaving threshold in the short-shallow reward environment and $D_{2/3}$ binding potential in the caudate nucleus (r=-0.2706, p=0.0871) and ventral striatum (r=-0.3061, p=0.0516). In addition, there was a positive trend between deviation from MVT optimal leaving threshold in the long-steep environment and dopamine synthesis capacity in the ventral striatum (r=0.2421, p=0.0973). None of these correlations held up to correction for multiple comparisons.

Body Mass Index (BMI) Association with Foraging Behavior and PET Measures

To assess for an impact of BMI on foraging behavior and PET measures, we extracted BMI data from the medical record. Forty-one participants had at least one BMI measure around the time of their PET scan. Specifically, forty-one participants had BMI data at the time of the [^{18}F]-FDOPA scan, 30 at the time of the [^{11}C]-NNC112 scan, and 29 at the time of the [^{18}F]-Fallypride scan. BMI values ranged from 19.1 to 33.9 with a median of 25.4. We calculated the mean BMI across all PET scans and included it in a linear regression model with the PCA component scores and behavioral measures of interest. For the total change in leaving threshold, we found that BMI was not associated with behavior ($p=0.659$) and the correlations with components 1 and 4 remained significant at $p<0.05$ with BMI included in the model as a covariate of no interest. Likewise, for the total change in reaction time, BMI was not correlated with behavior ($p=0.599$) and the correlations with components 1 and 4 remained significant at $p<0.05$ after controlling for BMI. Controlling for BMI in the ROI analyses did not change the correlations and trends with D_1 receptor availability or presynaptic synthesis capacity, but the correlations with $D_{2/3}$ receptor availability no longer met a trend level of significance. However, this is difficult to interpret because the number of subjects dropped from 43 to 29 due to not being able to obtain BMI measurements for all participants.

Temporal Stability of Results

There was a range of temporal separation between the behavioral task and PET scans; however, the median separation time was 9.00 months for [^{18}F]-FDOPA (interquartile range 47.5 months), 9.00 months for [^{18}F]-Fallypride (interquartile range 22.5 months), and 5.50 months for [^{11}C]-NNC112 (interquartile range 26.0 months). We ran sensitivity analyses controlling for the time between behavioral task and PET scan, which are included below. Furthermore, we tested the reliability of the behavioral measure using data collected from an independent sample.

Test-Retest Reliability of Foraging Behavioral Measures

In an experiment using a similar foraging paradigm that modulated travel time but not decay rate, 20 healthy adults without neuropsychiatric illness (mean age 61 years, 11 women) completed the patch-foraging task at two visits, approximately a week apart[1]. We obtained the data from this experiment from our collaborator, Sara Constantino, and tested the stability of our measures of interest. Both change in exit threshold and change in reaction time were consistent across testing dates (change in exit threshold: r=0.599, p=5.22e-3; change in reaction time: r=0.774, p=6.07e-5; see Supplementary Figure 8). Furthermore, the mean exit threshold in the individual reward environments were also stable across testing sessions (short: r=0.589, p=6.28e-3; long: r=0.599, p=5.27e-3). While the temporal separation between the PET scans and behavioral task for some subjects in our study was quite substantial, it is reassuring that these measures have been shown to be consistent across repeated testing sessions.

Future studies are needed to assess longer-term stability of foraging behavioral adjustments.

ROI Results Controlling for Time Between Behavioral Task and PET Scan

To investigate whether the ROI results were affected by variable amounts of time elapsed between the PET scans and the foraging task, we performed partial correlation analyses including time as an additional covariate of no interest. Adding this covariate did not change our results. We still found a positive correlation between total change in leaving threshold and the same PET measures: D_1 receptor availability positive correlation in the ventral striatum (r=0.399, p=0.0088) with trends in the ACC (r=0.295, p=0.0582) and putamen (r=0.299, p=0.0545); $D_{2/3}$ receptor availability positive trends in the putamen (r=0.295, p=0.0766), caudate nucleus (r=0.302, p=0.0693), and ventral striatum (r=0.310, p=0.0619); presynaptic dopamine synthesis capacity trend in the ACC (r=0.246, p=0.0954). We also saw the same results for change in reaction time: D_1 receptor availability positive correlation in the ventral striatum (r=0.476, p=0.0014), caudate nucleus (r=0.413, p=0.0066), putamen (r=0.317, p=0.0411), and ACC (r=0.327, p=0.0346); $D_{2/3}$ receptor availability positive correlation in the ventral striatum (r=0.335, p=0.0427) with trend in the putamen (r=0.298, p=0.0732); presynaptic dopamine synthesis capacity positive correlation in the midbrain (r=0.408, p=0.0045) with trends in the ACC (r=0.252, p=0.0881) and ventral striatum (r=0.255, p=0.0831).

Reliability of PET PCA Results

To assess the stability of the PCA solution, we used two approaches. First, we examined an independent sample of 26 individuals who had also completed all three PET scans (age 18-49 years, 12 females). Second, we used a bootstrapping sampling approach (with 1000 iterations) of the combined sample of our original subjects and the additional 26 subjects (total = 63 subjects). We drew random samples of participants with group size 37 (to match the original PCA analysis group) and calculated the confidence interval for the correlation coefficients for each component. We found that component 1 was stable in both the replication and bootstrapping analyses (correlation between component 1 coefficient in original sample and replication sample: r=0.702, p=0.0051; 95% confidence interval for the correlation coefficient of component 1 coefficient=0.674-0.708). The other components had lower correlations between samples, although the correlation with component 2 was significant at a threshold of p<0.05 (component 2: replication sample r=-0.5592, p=0.0376, bootstrapping 95% CI for the absolute value of the correlation coefficient=0.518-0.554; component 3: replication sample r=0.169, p=0.5626, bootstrapping 95% CI for the absolute value of the correlation coefficient=0.518-0.554; component 4: replication sample r=0.0669, p=0.820, bootstrapping 95% CI for the absolute value of the correlation coefficient=0.3749-0.4053).

Supplementary Figures

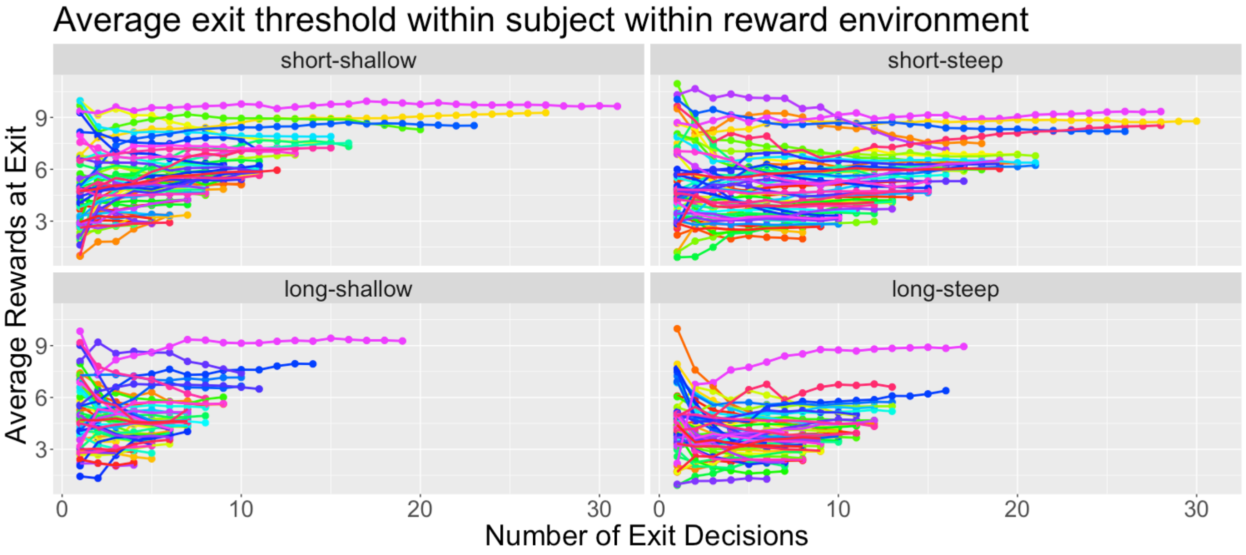

Supplementary Figure 1: **Moving average reward patch exit threshold over exit decisions, grouped by current reward environment.** Each color represents an individual participant (n=56). Source data are provided as a Source Data file.

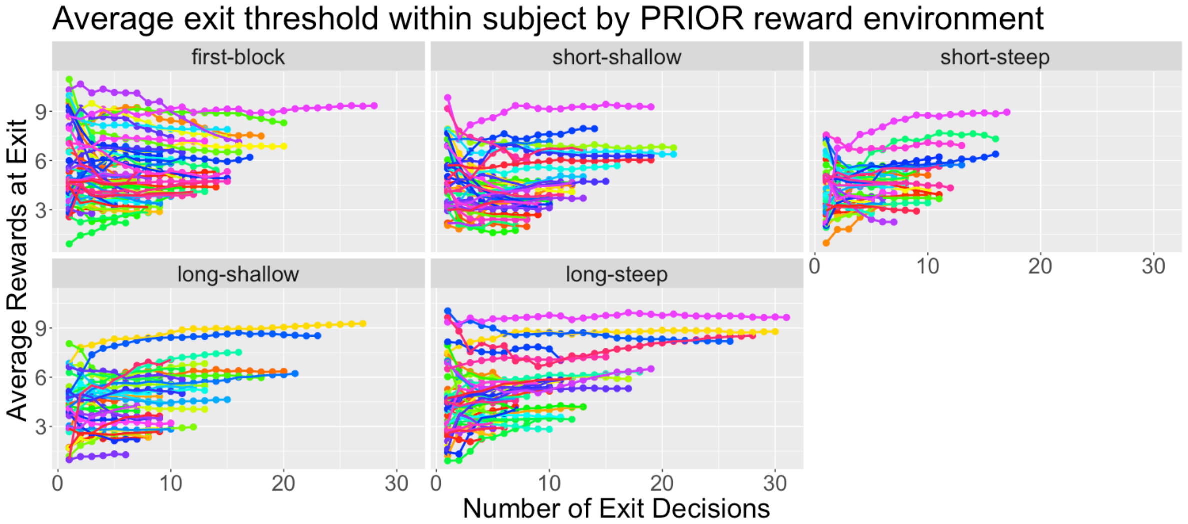

Supplementary Figure 2: **Moving average reward patch exit threshold over exit decisions, grouped by previous reward environment.** Each color represents an individual participant (n=56). Note that first-block indicates that the plotted environment was the first block of the experiment. Source data are provided as a Source Data file.

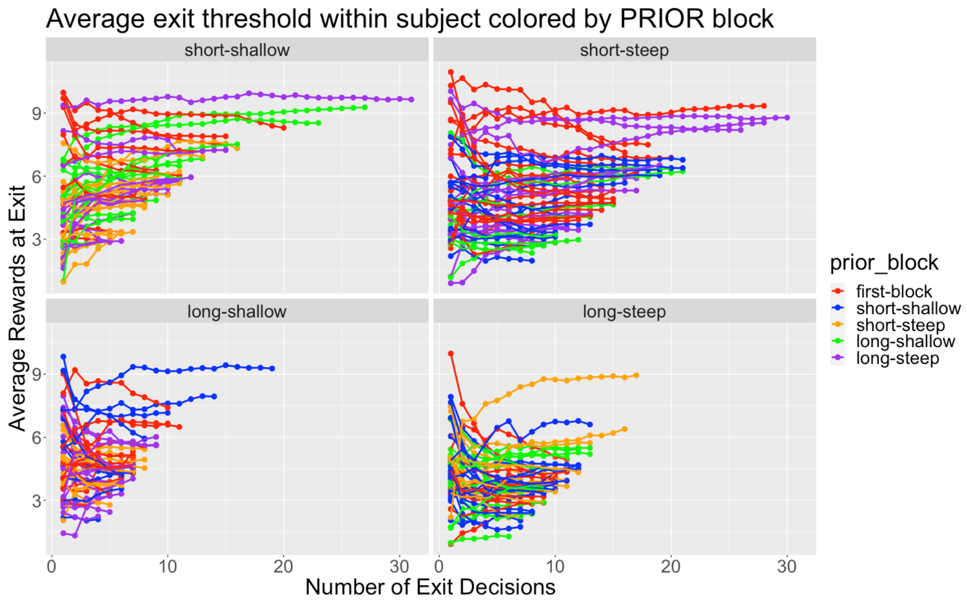

Supplementary Figure 3: **Moving average reward patch exit threshold over exit decisions, grouped by current reward environment and colored by prior block that the participant encountered.** Each line represents an individual participant (n=56) and color indicates the prior reward environment that the participant completed, defined in the figure legend. Note that first-block indicates that the plotted environment was the first block of the experiment. Source data are provided as a Source Data file.

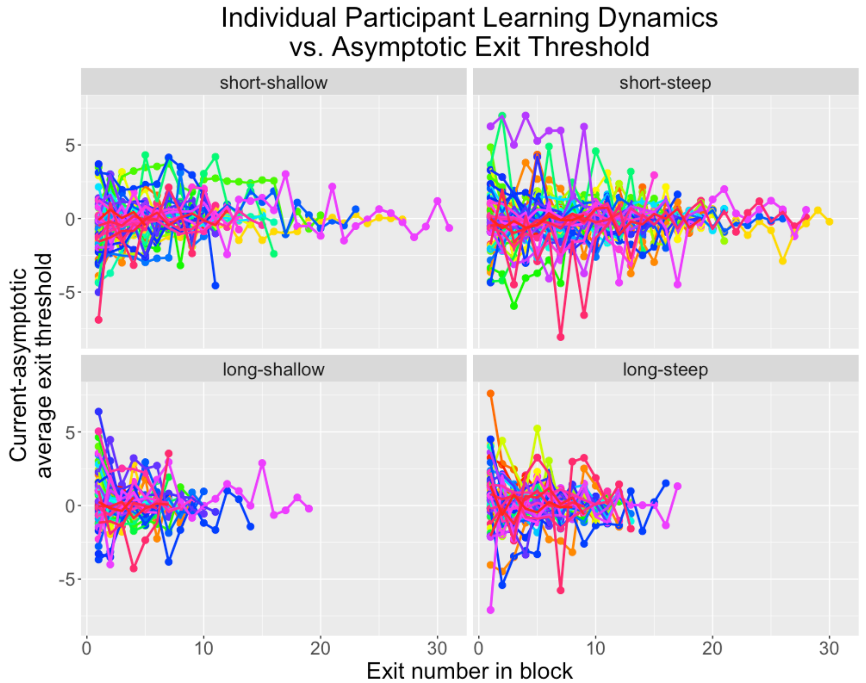

Supplementary Figure 4: **Difference between current and asymptotic (average of last three exit decisions) exit threshold within each reward patch, grouped by current reward environment.** Each color represents an individual participant (n=56). Source data are provided as a Source Data file.

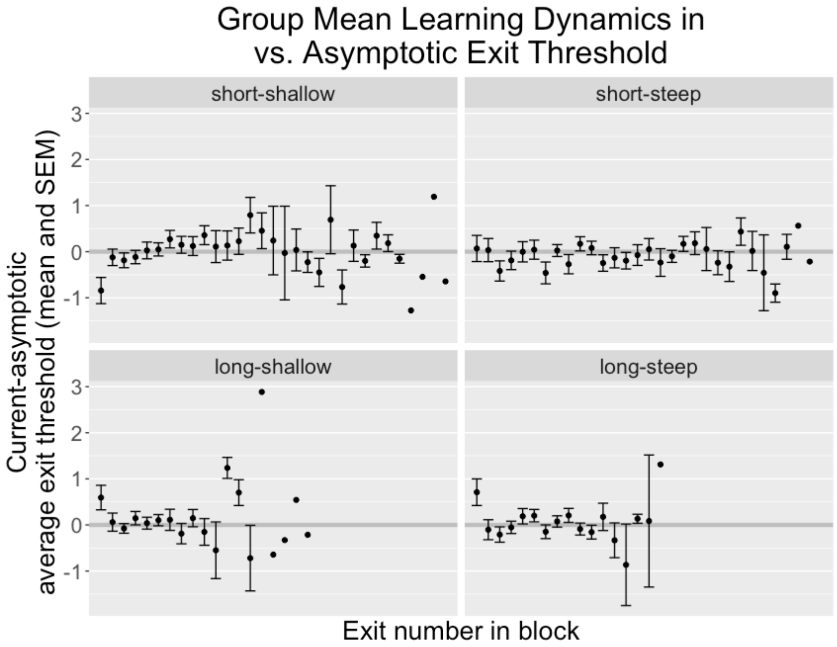

Supplementary Figure 5: **Group mean and standard error of the mean (SEM) of the difference between current and asymptotic (average of last three exit decisions) exit threshold for each exit decision, grouped by current reward environment (block).** Data are shown for n=56 individual participants. Individual data points are shown in Supplementary Figure 4. Source data are provided as a Source Data file.

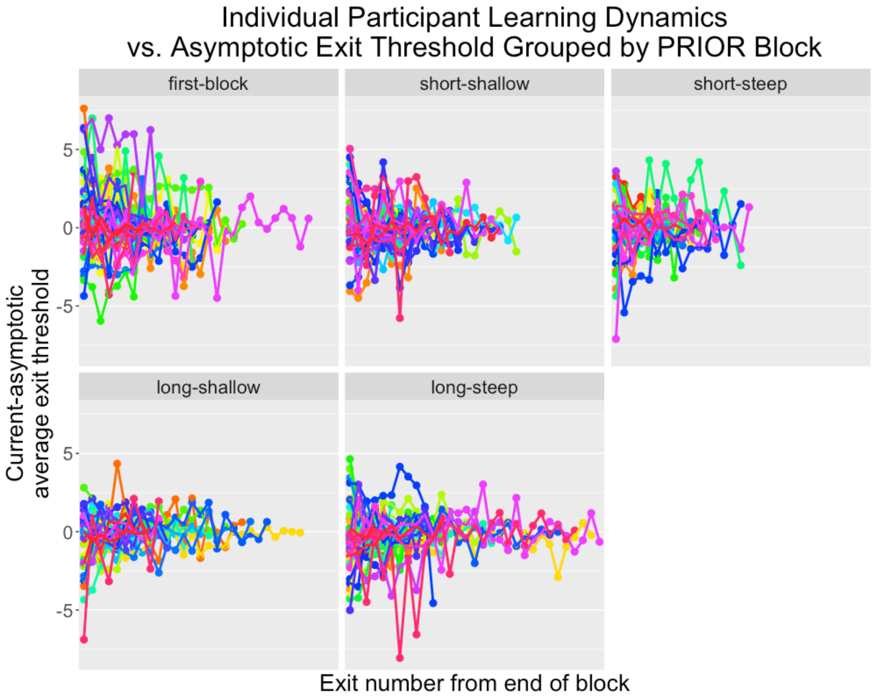

Supplementary Figure 6: **Difference between current and asymptotic (average of last three exit decisions) exit threshold for each exit decision, group by prior reward environment that the participant experienced.** Each color represents an individual participant (n=56). Source data are provided as a Source Data file.

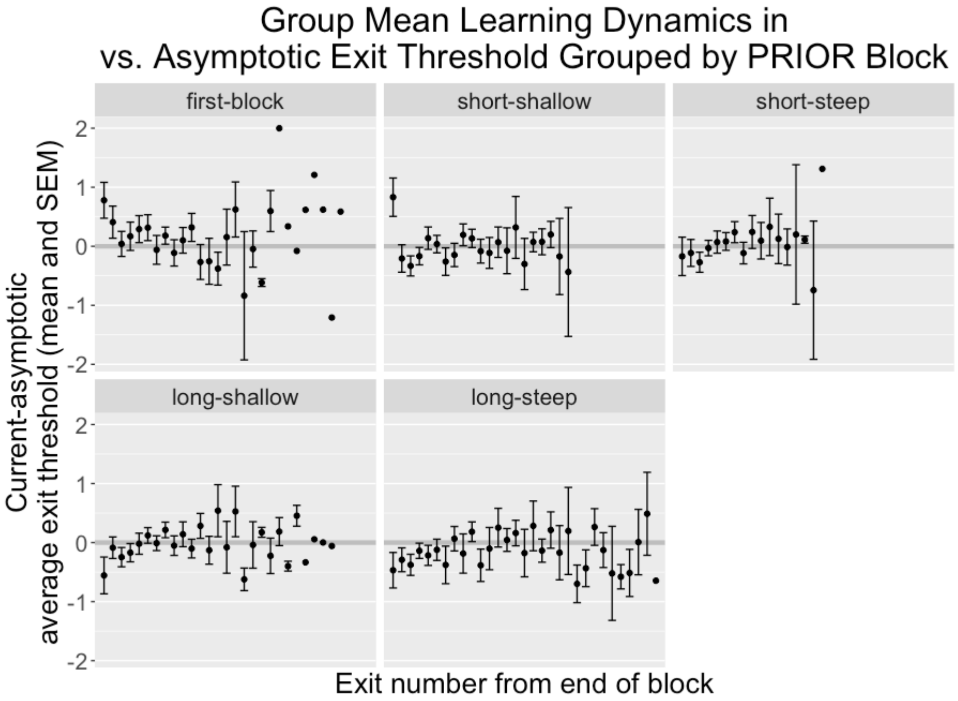

Supplementary Figure 7. **Group mean and standard error of the mean (SEM) of the difference between current and asymptotic (average of last three exit decisions) exit threshold, grouped by prior reward environment that the individual encountered.** Data are shown for n=56 individual participants. Individual data points are shown in Supplementary Figure 6. Source data are provided as a Source Data file.

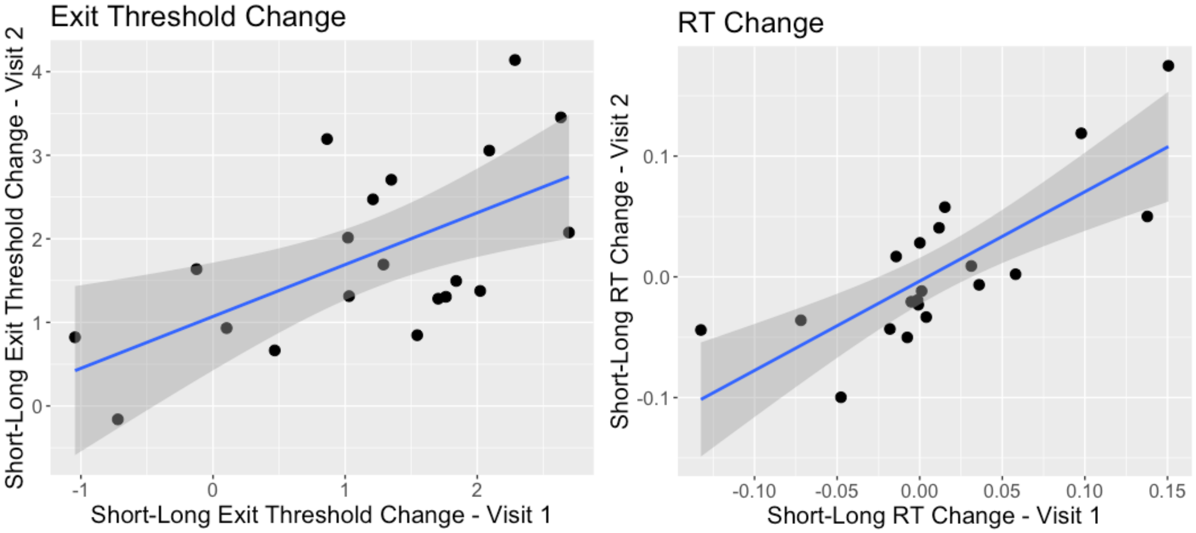

Supplementary Figure 8: **Stability of change in exit threshold and change in reaction time across two testing dates.** Change in exit threshold between visits 1 and 2 had a correlation coefficient of r=0.599 (p=5.22e-3, n=20 individual participants) and change in reaction time between visits 1 and 2 had a correlation coefficient of r=0.774 (p=6.07e-5). Source data are provided as a Source Data file.
