# Supplementary material for: PET-measured human dopamine synthesis capacity and receptor availability predict trading rewards and time-costs during foraging
Source: Nat Commun. 2023 Sep 30;14:6122. doi: 10.1038/s41467-023-41897-0 (PMC10542376; doi:10.1038/s41467-023-41897-0)
Supplement: Supplementary file 3 — Reporting Summary [file 41467_2023_41897_MOESM3_ESM.pdf]

nature portfolio

Corresponding author(s): Angela Ianni

Last updated by author(s): Aug 30, 2023

Reporting Summary

Nature Portfolio wishes to improve the reproducibility of the work that we publish. This form provides structure for consistency and transparency in reporting. For further information on Nature Portfolio policies, see our Editorial Policies and the Editorial Policy Checklist.

Statistics

For all statistical analyses, confirm that the following items are present in the figure legend, table legend, main text, or Methods section.

n/a	Confirmed	
☐	☒	The exact sample size (*n*) for each experimental group/condition, given as a discrete number and unit of measurement
☐	☒	A statement on whether measurements were taken from distinct samples or whether the same sample was measured repeatedly
☐	☒	The statistical test(s) used AND whether they are one- or two-sided<br>*Only common tests should be described solely by name; describe more complex techniques in the Methods section.*
☐	☒	A description of all covariates tested
☐	☒	A description of any assumptions or corrections, such as tests of normality and adjustment for multiple comparisons
☐	☒	A full description of the statistical parameters including central tendency (e.g. means) or other basic estimates (e.g. regression coefficient) AND variation (e.g. standard deviation) or associated estimates of uncertainty (e.g. confidence intervals)
☐	☒	For null hypothesis testing, the test statistic (e.g. *F*, *t*, *r*) with confidence intervals, effect sizes, degrees of freedom and *P* value noted<br>*Give P values as exact values whenever suitable.*
☒	☐	For Bayesian analysis, information on the choice of priors and Markov chain Monte Carlo settings
☒	☐	For hierarchical and complex designs, identification of the appropriate level for tests and full reporting of outcomes
☒	☐	Estimates of effect sizes (e.g. Cohen's *d*, Pearson's *r*), indicating how they were calculated

Our web collection on statistics for biologists contains articles on many of the points above.

Software and code

Policy information about availability of computer code

Data collection	The behavioral task code, implemented in Python, was obtained from co-author Dr. Sara Constantino and can be provided upon request. Task details are additionally available at (PMID: 25917000, DOI: 10.3758/s13415-015-0350-y).
Data analysis	Custom Matlab code was used for behavioral and PCA analyses, implemented in MATLAB R2021a (https://www.mathworks.com/products/matlab.html). The code is publicly available on Github (https://github.com/angmirian/Foraging_Dopamine_2023; Zenodo DOI 10.5281/zenodo.8283106). PET data were prepared with the following publicly available software: Freesurfer (https://surfer.nmr.mgh.harvard.edu/), FSL (http://fsl.fmirb.ox.ac.uk/fsl/), ANTS (http://stnava.github.io/ANTs/), and PMOD (http://www.pmod.com/web/).

For manuscripts utilizing custom algorithms or software that are central to the research but not yet described in published literature, software must be made available to editors and reviewers. We strongly encourage code deposition in a community repository (e.g. GitHub). See the Nature Portfolio guidelines for submitting code & software for further information.

Data

Policy information about availability of data

All manuscripts must include a data availability statement. This statement should provide the following information, where applicable:

- Accession codes, unique identifiers, or web links for publicly available datasets
- A description of any restrictions on data availability
- For clinical datasets or third party data, please ensure that the statement adheres to our policy

The data generated in this study are provided in the Source Data file accompanying this publication. Additional data are available upon request from the corresponding author (Angela Ianni, ianniam@upmc.edu). Requests must be consistent with individual participant consent and, as appropriate, may be subject to review by the NIH Internal Review Board. Source data are provided with this paper.

Research involving human participants, their data, or biological material

Policy information about studies with human participants or human data. See also policy information about sex, gender (identity/presentation), and sexual orientation and race, ethnicity and racism.

Reporting on sex and gender	The study includes sex as self-reported by participants to the research team. The sample is made up of 29 females and 28 males. The results reported apply to the consolidated group of all sexes, with sex included as a control covariate in statistical analyses. We did not conduct analyses for sexes individually as this was not part of the study design.
Reporting on race, ethnicity, or other socially relevant groupings	Self-reported race was African American (5 participants), Arabic (1 participant), Asian (1 participant), and Caucasian (50 participants). The results reported apply to the consolidated group of all races. We did not conduct analyses for individual effects of race as this was not part of the study design.
Population characteristics	See details in Behavioral & Social Sciences Study Design section below.
Recruitment	Participants were recruited from the local (i.e. Bethesda, Maryland and surrounding area) community. Participants were required to be between 18 and 60 years of age and no participants were excluded based on race (sample contained ~10% African American, consistent with the surrounding community). Participant group could contain self-selection bias for those individuals with sufficient time and motivation to participate in research.
Ethics oversight	All studies were completed at the National Institutes of Health Clinical Center and were approved by the NIH Combined Neuroscience Institutional Review Board and the National Institutes of Health Radiation Safety Committee.

Note that full information on the approval of the study protocol must also be provided in the manuscript.

Field-specific reporting

Please select the one below that is the best fit for your research. If you are not sure, read the appropriate sections before making your selection.

☐ Life sciences ☒ Behavioural & social sciences ☐ Ecological, evolutionary & environmental sciences

For a reference copy of the document with all sections, see nature.com/documents/nr-reporting-summary-flat.pdf

Behavioural & social sciences study design

All studies must disclose on these points even when the disclosure is negative.

Study description	Quantitative cross-sectional study of healthy adults who completed a behavioral foraging task, MRI, as well as three PET scans on separate days.
Research sample	The sample consists of 57 adult healthy volunteers, aged 21-57 years with 29 females and 28 males. The sample is representative of Bethesda, Maryland and surrounding area. Participants were screened by clinician-administered history and exam as well as structural MRI read by a neuroradiologist to exclude confounding medical and psychiatric disorders.
Sampling strategy	Participants were sampled from ongoing recruitment for the 09M0176/NCT00942981 and 01M0232/NCT00024622 protocols. We ran a power analysis based on our multiple regression analysis for the primary result of component 1 and 4 scores being correlated with total change in leaving threshold. The r-squared value for that regression with four predictors was 0.295. This corresponds to an effect size (f-squared) of 0.418. Power calculation revealed that for a power level of 0.8, 35 subjects would be required and our experiment included 37 individuals who completed all three PET scans and were included in the PCA-based analyses.
Data collection	Behavioral data were collected on a Mac computer. Imaging data were collected on GE Advance 3D scanner (PET 18F-FDOPA), Siemens High-Resolution Research Tomograph (HRRT) scanner (18F-Fallypride and 11C-NNC112), and GE 3T MRI scanner. A researcher was present during behavioral data collection. Blinding was not applicable to the study design. PET data were collected by NIH Clinical Center PET technicians.

Timing	Participants were recruited from the ongoing PET protocols (09M0176 and 01M0232) between January 2014 and February 2016.
Data exclusions	One participant was excluded due to behavior that suggested they were not following the foraging task instructions (absence of any leave decisions in one of the blocks of the task). 18F-FDOPA putamen ROI data was excluded for one participant due to having a value that was more than three standard deviations from the mean.
Non-participation	There were no participants that dropped out of the study. All 57 participants completed the foraging behavioral task. Of these participants, 51 completed the 18F-FDOPA PET scan, 45 completed the 11C-NN112 PET scan, and 42 completed the 18F-Fallypride PET scan.
Randomization	For the foraging behavioral task, the order of task blocks was randomly assigned across participants.

Reporting for specific materials, systems and methods

We require information from authors about some types of materials, experimental systems and methods used in many studies. Here, indicate whether each material, system or method listed is relevant to your study. If you are not sure if a list item applies to your research, read the appropriate section before selecting a response.

Materials & experimental systems

n/a	Involved in the study
☒	☐ Antibodies
☒	☐ Eukaryotic cell lines
☒	☐ Palaeontology and archaeology
☒	☐ Animals and other organisms
☐	☒ Clinical data
☒	☐ Dual use research of concern
☒	☐ Plants

Methods

n/a	Involved in the study
☒	☐ ChIP-seq
☒	☐ Flow cytometry
☐	☒ MRI-based neuroimaging

Clinical data

Policy information about clinical studies

All manuscripts should comply with the ICMJE guidelines for publication of clinical research and a completed CONSORT checklist must be included with all submissions.

Clinical trial registration	ClinicalTrials.gov identifiers for the data included in this manuscript include: NCT00004571, NCT00942981, and NCT00024622
Study protocol	Data were collected under NIH protocols: 00M0085/NCT00004571, 09M0176/NCT00942981, and 01M0232/NCT00024622
Data collection	As noted above, participants were recruited from the ongoing PET protocols (09M0176 and 01M0232) between January 2014 and February 2016.
Outcomes	There were no clinical outcomes collected as part of this study. Participants were all healthy volunteers.

Magnetic resonance imaging

Experimental design

Design type	Structural MRI only
Design specifications	1 or more image, averaged together
Behavioral performance measures	NA; see above for foraging task details

Acquisition

Imaging type(s)	structural
Field strength	3T
Sequence & imaging parameters	Specific sequence parameters varied as a subject-specific template was created for each participant using the available structural MRI images. Most images used the MPRAGE sequence.
Area of acquisition	whole brain

Diffusion MRI ☐ Used ☒ Not used

Preprocessing

Preprocessing software	We used the following software for preprocessing: Freesurfer (https://surfer.nmr.mgh.harvard.edu/) for MRI segmentation, FSL FLIRT (http://fsl.fmirb.ox.ac.uk/fsl/) for inter-scan motion correction and realignment of PET images, ANTS (http://stnava.github.io/ANTs/) spatial normalization of MRI and PET data, and PMOD (http://www.pmod.com/web/) for PET data modeling.
Normalization	For analyses of basal ganglia ROIs, no normalization was used, as ROI tracer data were extracted from native space segmentations. For MNI-space derived anterior cingulate/voxelwise calculations, diffeomorphic exponential normalization as implemented in ANTS was used for spatial warping.
Normalization template	MNI-space
Noise and artifact removal	FSL-FLIRT was used for inter-scan motion correction.
Volume censoring	N/A

Statistical modeling & inference

Model type and settings	General linear model
Effect(s) tested	Association between PET PCA component scores and foraging behavioral measures

Specify type of analysis: ☐ Whole brain ☒ ROI-based ☐ Both

Anatomical location(s)	Regions of interest (ROIs) included dorsal putamen, dorsal caudate nucleus, ventral striatum, and dopaminergic midbrain, which were generated using Freesurfer segmentation. Anterior cingulate cortex ROI was created as a 5mm-radius sphere centered on the peak voxel encoding foraging average reward value from the prior Kolling et al, Science, 2012.
Statistic type for inference (See Eklund et al. 2016)	Mean ROI values were normalized and entered into the principal component analysis. Statistics were applied to whole-ROI principal components.
Correction	FDR (whole-ROI)

Models & analysis

n/a	Involved in the study
☒	☐ Functional and/or effective connectivity
☒	☐ Graph analysis
☒	☐ Multivariate modeling or predictive analysis
